# Identifying Direct Coercion in a High Risk Subgroup of Offender Patients With Schizophrenia *via* Machine Learning Algorithms

**DOI:** 10.3389/fpsyt.2020.00415

**Published:** 2020-05-13

**Authors:** Moritz Philipp Günther, Johannes Kirchebner, Steffen Lau

**Affiliations:** ^1^Department of Psychiatry, Psychotherapy and Psychosomatics, University Hospital of Psychiatry Zurich, Zurich, Switzerland; ^2^Department of Forensic Psychiatry, University Hospital of Psychiatry Zurich, Zurich, Switzerland

**Keywords:** coercion, seclusion, restraint, involuntary medication, offenders with schizophrenia spectrum disorder, severe mental illness, machine learning, forensic psychiatry

## Abstract

**Purpose:**

This study aims to explore risk factors for direct coercive measures (seclusion, restraint, involuntary medication) in a high risk subpopulation of offender patients with schizophrenia spectrum disorders.

**Methods:**

Five hundred sixty nine potential predictor variables were explored in terms of their predictive power for coercion/no coercion in a set of 131 (36.6%) offender patients who experienced coercion and 227 who did not, using machine learning analysis. The dataset was split (70/30%) applying variable filtering, machine learning model building, and selection embedded in nested resampling approach in one subset. The best model was then selected, and the most important variables extracted on the second data subset.

**Results:**

In the final model the following variables identified coercion with a balanced accuracy of 73.28% and a predictive power (area under the curve, AUC) of 0.8468: threat of violence, (actual) violence toward others, the application of direct coercive measures during past psychiatric inpatient treatments, the positive and negative syndrome scales (PANSS) poor impulse control, uncooperativeness, and hostility and the total PANSS-score at admission, prescription of haloperidol during inpatient treatment, the daily cumulative olanzapine equivalent antipsychotic dosage at discharge, and the legal prognosis estimated by a team of licensed forensic psychiatrists.

**Conclusions:**

Results confirm prior findings, add detail on factors indicative for the use of direct coercion, and provide clarification on inconsistencies. Limitations, clinical relevance, and avenues for future research are discussed.

## Introduction

For a uniform definition of direct (formal, institutional) coercive measures in psychiatry it has been proposed to encompass restraint, seclusion, and involuntary medication (1–3): restraint is to include physical restraint by another person or mechanical restraint with a device, seclusion is to involve the locking up of a person alone in a room, and involuntary medication encompasses the administration of medication against a patient's will. Various guidelines and associations of professionals in mental health care have long called for a reduction in the use of such practices for numerous reasons including legal, economic, and ethical concerns, doubts in their effectiveness and worries over short- and long-term effects of such measures on patients', professionals', and their social network's physical and mental health (4–7). Prior research has identified numerous risk factors for seclusion (8–14), restraint (15–20), involuntary medication (21, 22), or combinations thereof (16, 23–35) with differences and inconsistencies in reported predictor variables being larger between studies exploring the same coercive measure than between coercive measures. The most frequently identified predictors include schizophrenia spectrum disorder (8, 12, 17, 19, 22, 25, 27, 30, 31, 34, 35), a threat of violence/aggression (9, 18, 20, 24, 25, 36–40), prior involuntary (admission to) treatment (15, 17, 19, 22, 27, 30, 34, 35), female gender (8, 11, 13, 20), male gender (10, 14, 16, 17, 19, 24, 25, 30, 33), younger age (10, 13, 14, 16–18, 20, 24, 25, 33, 34), older age (19), and substance abuse (8, 34). Moreover, there is no consensus on whether restraint, seclusion, or involuntary medication are more intrusive and detrimental on patients' well-being and they are often used in combination or as a partial or complete substitute for each other depending on cultural norms or legal statutes prohibiting the use of one or another thus resulting in skewed results if only one measure of coercion is explored (2, 3, 23, 26, 37, 41). Another confounder in research on the prevalence of direct coercive measures in general psychiatry stems from some patients with behavior resulting in the frequent use of coercive measures but no criminal history being nonetheless treated in forensic psychiatry in some countries but not others (3, 42). There is a general consensus that more research on the use of coercive measures in forensic psychiatry is needed (1, 3) in an era in which the caring aspect of forensic treatment has achieved equality in comparison to custodial objectives (i.e., public safety). Furthermore, particularly subtle factors contributing to the use of coercive measures in psychiatry as a whole may be more pronounced and observable in populations of patients with high risk for coercive measures, which can be found in forensic psychiatry (3). With predictors of coercive measures identified so far being mostly broad categories, research identifying finer, more specific predictors for coercion is needed.

Two recent studies used machine learning to explore factors predicting the use of any direct coercive measures (30) or mechanical restraint in particular (15) in general psychiatry. With machine learning being developed to reveal previously “unseen” non-linear interdependencies between variables (43, 44), both studies were enabled to analyze a much greater number of potential predictors and identify more detailed clinically relevant factors predicting coercion with better model performance and generalizability (due to cross-validation) than prior research using conventional statistical procedures (15, 30). Both recognize a need for similar research in other treatment settings and recommend machine learning due to its results' superiority to those from contemporary statistical techniques in terms of their generalizability, sensitivity, specificity, accuracy, and predictive validity (AUC: area under the curve).

The purpose of the current study is to employ machine learning for the analysis of 569 potential predictors of direct coercive measures in 370 Swiss forensic offender patients with a schizophrenia spectrum disorder during their involuntary inpatient treatment. By selecting a sample in which the presence of those factors most frequently and consistently identified to predict coercive measures (schizophreniform disorder, threats of violence, involuntary admission) are present in all cases, we aim to identify more subtle and detailed predictors of coercion.

## Materials and Methods

### Source of Data

In our retrospective study design, directed qualitative content analysis (45) was used to extract data from files of 370 offender patients with a schizophrenia spectrum disorder according to ICD-10 (46) judicially admitted to the Centre for Inpatient Forensic Therapies at the Zurich University Hospital of Psychiatry between 1982 and 2016. Specifically, over 500 parameters were rated with the extended (47, 48) rating protocol based on criteria proposed by Seifert (49) by one trained independent physician with subsequent validation by a second trained independent coder analyzing a random subsample of 10% of cases with a Cohen's Kappa of 0.78 indicating substantial inter-rater reliability (50, 51). In light of the legal requirements in Switzerland, files can be assumed to be composed with utmost care and included anamneses, psychiatric assessments (including psychopathology), past and current medication, and other treatments documented by licensed psychiatrists and psychologists trained in psychotherapy, reports from other trained health care professionals (nursing and care staff, social workers), police reports, testimonies, court proceedings, and other legal documents. Documentation of psychopathological symptoms was available from licensed psychiatrists diagnosing and treating patients prior to the index offense, forensic psychiatrists immediately after admission of offenders to the forensic center and before their discharge. A close adoption of the Positive and Negative Symptom Scale (PANSS) was used to categorize and quantify psychopathological symptoms (into 30 subcategories dichotomized to symptom not present/symptom present) during content analysis (52). Antipsychotic dosages per day after admission and at discharge were converted into olanzapine equivalents by using conversion factors provided through the classical weighted mean dose method (53) if possible and the minimum effective dose method (54) or (lastly) international experts' consensus based olanzapine equivalents (55) in all other instances.

Analysis of all cases included in this study was approved by the Zurich Cantonal Ethics Committee.

### Machine Learning

Supervised machine learning (ML) is suitable for explorative analyses such as in the current study. In ML, a so-called outcome variable is defined *a priori* (often dichotomous; e.g., coercion/no coercion) and numerous other variables are tested for their ability to predict this outcome. Thus, ML means a computer algorithm [such as logistic regression, support vector machines (SVM), decision trees or k-nearest neighbor (KNN) depending on the data structure] is developed which uses all variables available (e.g., psychopathology, medication, biography) in order to try predict the outcome variable for any given patient.

In contrast to conventional (hypothesis testing) statistical methods, ML is able to uncover hidden interrelationships in data sets, can explore a larger set of variables at once, and can use various (linear and non-linear) algorithms, which can be evaluated quantitatively by transcending p-value thresholds. One of the most significant risks in ML is overfitting. This means that the mathematical algorithms depend heavily on the data structure and are sensitive to “noise” within the data, which leads to overestimation in the prediction. Especially if the study population is small and many variables are explored, there is a substantial risk for overfitting. Techniques designed to minimize the risk for overfitting include splitting the data to obtain a separate set of data for testing ML-results, cross-validation of ML results, regularization, or a reduction of the (predictor) variables explored.

Despite such safeguards against overfitting, ML results from one data set should be treated with caution and need further confirmation by new data and perhaps more conservative statistical approaches before they can be considered to be generalizable (56–58).

### Statistical Analysis

**Figure 1:**
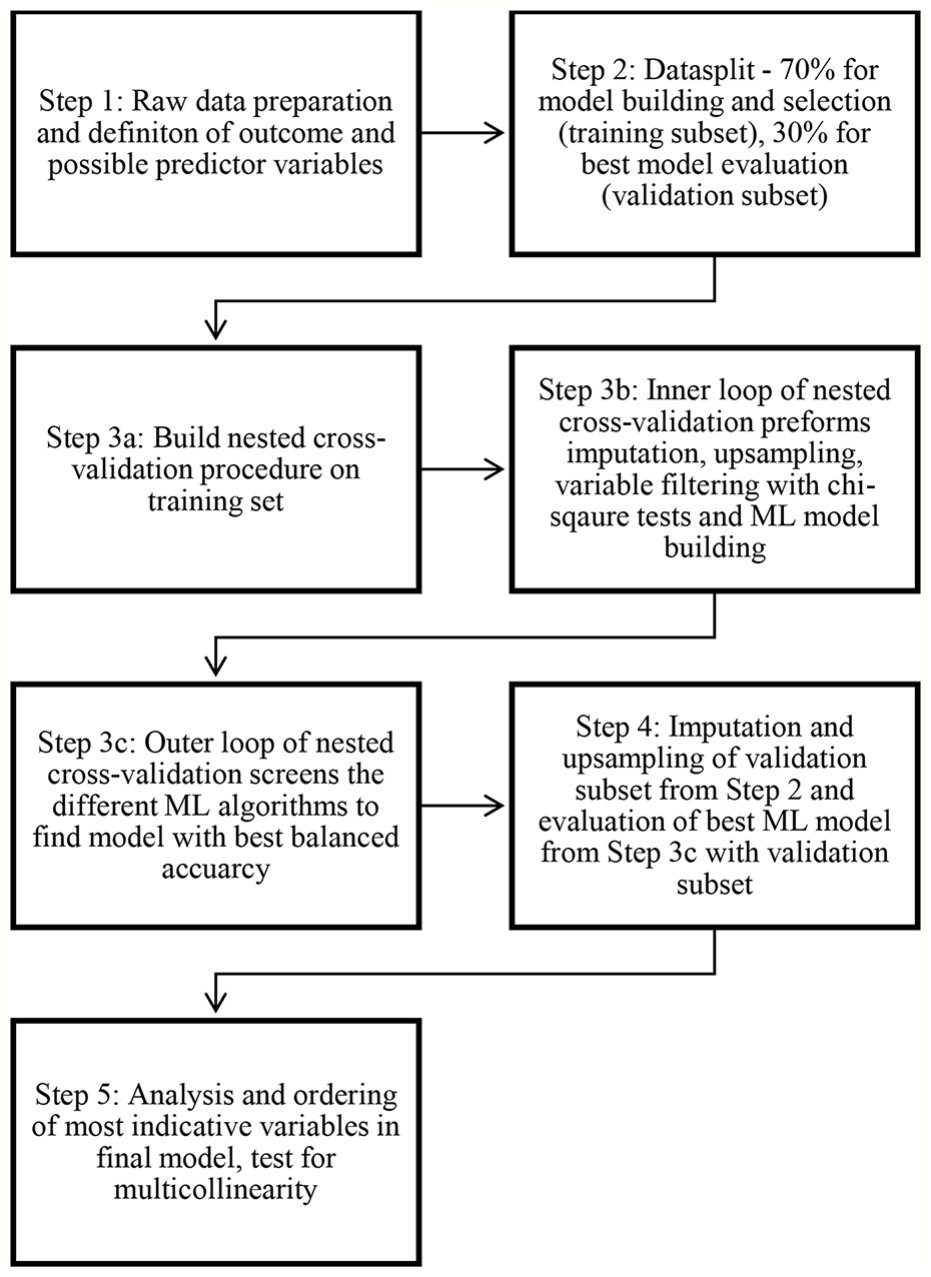
Data processing and statistical analysis.

**Figure 1** provides an overview of the statistical Steps taken in the present study, which are described in detail below. All Steps were performed using R version 3.6.3. and the MLR package v2.171 (59). CI calculations of the balanced accuracy were conducted using MATLAB R2019a (MATLAB and Statistics Toolbox Release 2012b, The MathWorks, Inc., Natick, Massachusetts, United States) with the add-on “computing the posterior balanced accuracy” v1.0 (60). Online calculators were used to obtain the CI auf AUC (61) and the CI of the remaining classification performance measures (62).

### Preliminary Data Processing and Measures

All raw data was first processed for machine learning (see **Figure 1** Step 1)—multiple categorical variables were converted to binary code. Continuous and ordinal variables were not manipulated. Variables with more than 33% missing values were eliminated resulting in a remaining set of 570 variables.

Data on the use or non-use of direct coercive measures was available for 358 of all explored offender patients. Of these 131 (36.6%) experienced one or more direct coercive measure, which corresponds with rates of coercion reported in extant literature, ranging from 21 to 59% of patients (63). The occurrence of any one direct coercive measure or combinations thereof was defined to be the outcome variable for further analysis (i.e., coercion/no coercion). No coercion was defined as the positive class, coercion as the negative class.

Most patients were subjected to a combination of two or more measures of direct coercion. Just 31 (8.66%) patients were subjected only to seclusion and 12 (3.35%) only to involuntary medication. Physical restraint is not used in Switzerland and mechanical restraint was always used in combination with seclusion and/or involuntary medication.

Next, the initial dataset was randomly divided into two subsets (see **Figure 1**, Step 2)—a training dataset with 70% of all patient cases (251 patients) and a validation dataset with 30% of cases (107 patients). The training data set was used for variable reduction and model building/selection (see **Figure 1**, Steps 3a–c) whereas the validation data set was used to evaluate the previously selected statistical model (see **Figure 1**, Steps 4 and 5). The selection of predictor variables/model building and model evaluation was based on different subsets of the available data in order to minimize the risk for overfitting.

### Imputation, Balancing, Variable Filtering, Statistical Model Building/Selection, and Nested Resampling

All of the following Steps under above heading where performed with the training data set (251 patients) only, while the data set for validation (107 patients) remained untouched:

One main objective of the present study was to identify the most important predictor variables from the multitude of 569 possible variables. In addition, a reduction of variables was intended to counteract overfitting and keep computing times in initial model building at an acceptable level. Therefore, chi square testing was applied to the initial 569 predictor variables to filter for their 10 most predictive variables for further model building (see **Figure 1**, Step 3b).

In addition, to enable ML to more flexibly employ statistical approaches that are sensitive to missing values, imputation by mean for continuous variables and imputation by mode for categorical variables was applied to the training data set to estimate missing values (see **Figure 1**, Step 3b). The imputation weights were saved to be applied later to the validation data set (see **Figure 1**, Step 5).

Since the distribution of the outcome coercion/no coercion was not balanced (36.6 *vs.* 63.4%) this also led to a disbalance in the calculated sensitivity/specificity of the models, so the smaller subset (coercion experienced) was oversampled by a rate of 2.

However, overfitting is an issue to be guarded against. Furthermore, to achieve reliable performance estimates, imputation and variable filtering should be embedded in a cross validation process and model building and evaluation should be kept separated (64, 65).

Nested resampling is best suited for this objective—in an inner loop data processing Steps and model training can be performed imbedded in cross-validation and then in an outer loop the performance of these models can be tested also embedded in cross-validation. In this study the nested resampling model (see **Figure 1**, Step 3a) was built with the inner loop preforming the imputation, oversampling, variable filtration, and model building within 5-fold cross-validation (see **Figure 1**, Step 3b) and the outer loop being used for performance evaluation also embedded in 5-fold-cross-validation (see **Figure 1**, Step 3c), a technique of artificially creating different subsamples of a data set (66). In cross-validation balanced accuracy was optimized for.

In order to select the final statistical model (see **Figure 1**, Step 3c), different ML algorithms—logistic regression, trees, random forest, gradient boosting, KNN (k-nearest neighbor), support vector machines (SVM), and naive Bayes [for a more detailed description see (44)]—were trained (see **Figure 1**, Step 3b). No hyperparameters were optimized. The default hyperparameters can be obtained from the **Supplementary Materials**. Finally the model performance of each model was calculated and assessed in terms of its balanced accuracy and goodness of fit (measured with the receiver operating characteristic, balanced curve area under the curve method, ROC balanced AUC) (67) (see **Figure 1**, Step 3c). Moreover, specificity, sensitivity, positive predictive value (PPV), and negative predictive value (NPV) were evaluated. The described nested resampling strategy was applied for all ML algorithms and the ML model with the best balanced accuracy was chosen for final model validation with the data set for validation (107 patients, see **Figure 1**, Step 4).

### Model Validation and Variable Importance

The validation subset of the total data (30%, 107 patients) was imputed with the stored weights from Step 3b by mean and mode. Then, the best previously identified model was applied to the data and again the performance measures of this final model were assessed (see **Figure 1**, Step 4). The variables used to predict the outcome variable (coercion/no coercion) in the final model were ordered by indicative power and tested for multicollinearity (see **Figure 1**, Step 5), as will be detailed in the *Results*.

## Results

Sociodemographic characteristics and legal justifications for the application of direct coercion are summarized below (**Table 1**).

**Table 1:** Sociodemographic characteristics of the studied sample and legal justifications for use of direct coercion.

Characteristics	Total n/N (%)	No coercion n/N (%)	Coercion n/N (%)
Male sex	333/358 (93)	208/227 (91.6)	125/131 (95.4)
Age at admission (mean, SD)	33.99 (10.191)	34.40 (10.128)	33.27 (10.298)
Native country Switzerland	160/358 (44.7)	105/227 (46.3)	55/131 (42)
Single (at offense)	288/352 (80.4)	181/222 (81.5)	107/130 (82.3)
Legal justification for use of direct coercion			
Endangerment of self			19/131 (14.5)
Threat of violence			74/131 (56.5)
Violence against others (physical)			52/131 (39.7)

SD, standard deviation; N, total study population; n, subgroup with characteristic.

The performance measures of all trained models during the nested resampling procedure on the initial training subset (70% of the total data set) can be seen in **Table 2** (for detailed results such as CI see **Supplementary Materials**). With a balanced accuracy of 77% naïve Bayes was identified as the best performing algorithm.

**Table 2:** Machine learning models and performance during nested resampling.

Statistical procedure	Balanced accuracy (%)	AUC	Sensitivity (%)	Specificity (%)	PPV (%)	NPV (%)
Logistic regression	75.13	0.85	71	80	85	62
Tree	71.55	0.79	76	67	79	63
Random forest	74.12	0.86	78	70	81	66
Gradient boosting	72.63	0.84	76	69	80	63
KNN	69.56	0.80	68	71	80	57
SVM	76.85	0.84	81	73	83	69
Naive Bayes	77.01	0.84	85	73	84	69

AUC, area under the curve (level of discrimination); PPV, positive predictive value; NPV, negative predictive value; KNN, k-nearest neighbors; SVM, support vector machines.

The 10 most indicative variables (code, description, and distribution) identified through chi square testing and subsequently used for model building can be withdrawn from **Table 3**.

**Table 3:** Absolute and relative distribution of relevant predictor variables.

Variable code	Variable description	Coercion experienced	No coercion experienced
R13a	Threat of violence during current inpatient treatment	83/129 (64.3)	30/221 (13.6)
R20a	Violence toward others during current inpatient treatment	62/131 (47.3)	12/227 (5.3)
PH12a	Direct coercive measure applied in past psychiatric inpatient treatment	89/111 (80.2)	61/192 (31.8)
PANSSH28	PANSS-adopted scale at admission: Poor impulse control	98/131 (74.8)	83/224 (37.1)
PANSSH22	PANSS-adopted scale at admission: Uncooperativeness	97/131 (74)	90/224 (40.2)
R8a	Haloperidol prescribed during current inpatient treatment	72/130 (55.4)	57/225 (25.3)
PANSS SCORE ADMH (mean, SD)	Total PANSS score at admission	21.47 (13.03)	17.84 (14.47)
R9e (mean, SD)	Olanzapine equivalent dose at discharge	52.28 (17.83)	42.29 (19.98)
PANSSH7	PANSS-adopted scale at admission: Hostility	84/131 (64.1)	81/224 (36.2)
R28	Estimated legal prognosis		
	Favorable	18/110 (16.4)	53/200 (26.5)
	Sufficient	16/110 (14.5)	62/200 (31)
	Doubtful	27/110 (24.5)	35/200 (17.5)
	Unfavorable	49/110 (44.5)	50/200 (25)

SD, standard deviation; PANSS, positive and negative syndrome scale.

The final naïve Bayes model using these variables applied to the validation subset (30% of the total data set) yielded a balanced accuracy of 73.28% and an AUC of 0.8468 (see **Table 4**). This model had a sensitivity of 72.87%, reflecting its ability to correctly classify the actual cases “not having experienced coercion,” and a slightly higher specificity of 73.68%, indicating its ability to correctly identify those having “experienced coercion.”

**Table 4:** Final naïve Bayes model performance measures.

Performance measures	% (95% CI)
**Balanced accuracy**	73.28 (0.8272–0.5888)
**AUC**	0.8468 (0.9573–0.7363)
**Sensitivity**	72.87 (88.13–50.44)
**Specificity**	73.68 (88.21–52.26)
**PPV**	71.82 (87.33–49.57)
**NPV**	74.68 (88.96–53.12)

AUC, area under the curve (level of discrimination); PPV, positive predictive value; NPV, negative predictive value; CI, confidence interval.

**Figure 2:**
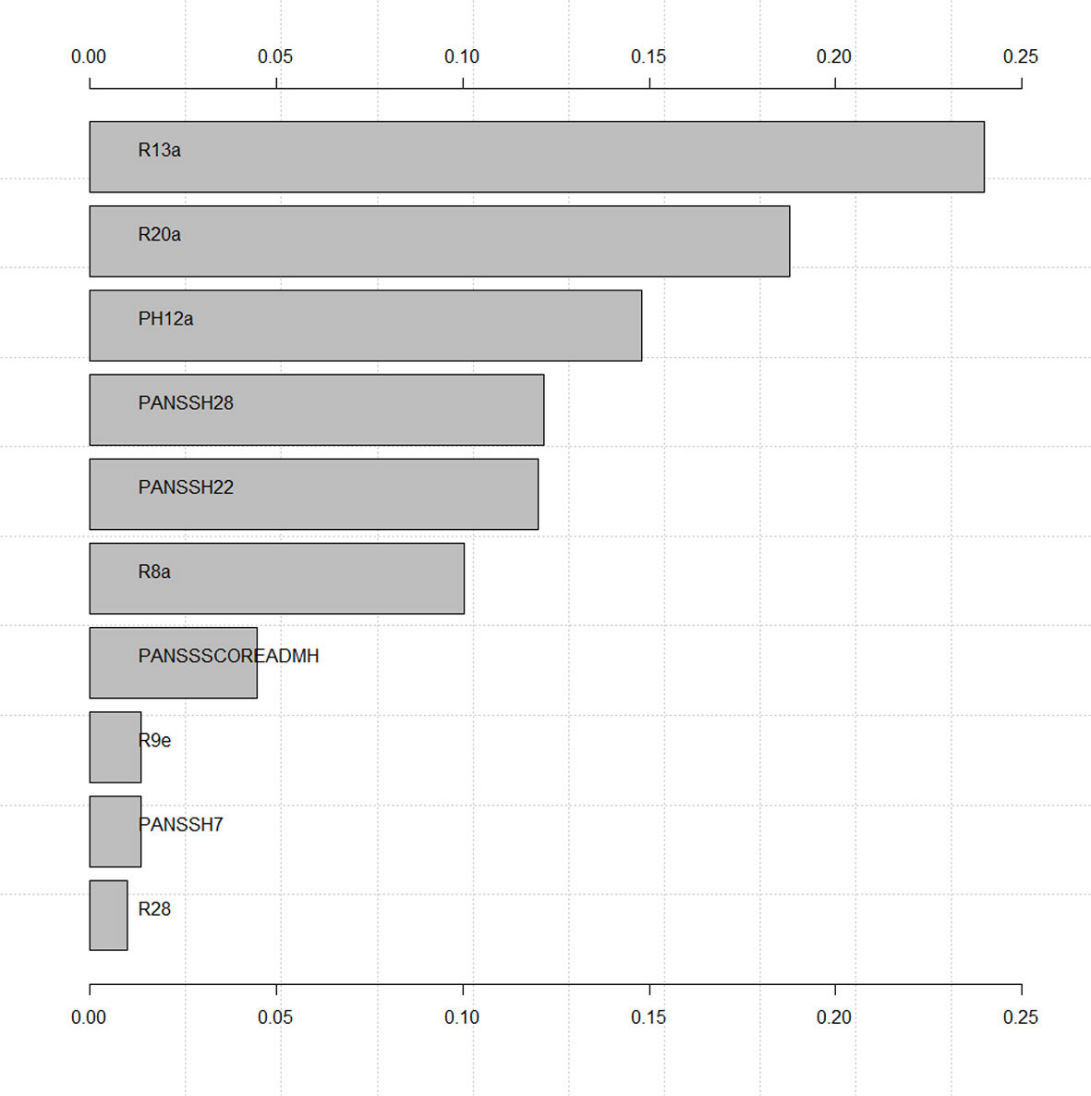
Variable importance of final model. Variable descriptions are presented in **Table 3**.

Testing for multicollinearity showed no dependencies between the variables (detailed results see **Supplementary Materials**). The importance of each variable in the naïve Bayes model can be seen in **Figure 2**. Threat of violence and actual violence were identified as most indicative factors for coercion. Past experiences with coercion was the 3^rd^ most indicative factor. The PANSS scales at admission poor impulse control and uncooperativeness leading to a higher total PANSS score were also identified as influential factors for the model as well as experiences with haloperidol during the current hospitalization. The olanzapine equivalent dose at discharge, the PANSS scale hostility at admission and the estimated legal prognosis of the patient (evaluated by a board of forensic psychiatrists before discharge) were least important for the final model.

## Discussion

Machine learning was used to identify patients who experienced direct coercive measures (seclusion, restraint, involuntary medication) in a set of 358 offender patients with a schizophrenia spectrum disorder during forensic psychiatric inpatient treatment. The best identifiers out of a set of 569 potential variables were (in order of statistical significance): threat of violence and actual violence toward others during inpatient treatment, direct coercive measures in the past, poor impulse control and uncooperativeness at admission, the prescription of haloperidol during inpatient treatment, the total PANSS-score at admission, the daily cumulative olanzapine equivalent antipsychotic dosage at discharge, hostility at admission, and the legal prognosis as estimated by a team of forensic psychiatrists upon discharge based on all available information in a patient's file. Based on these variables the model was able to predict the occurrence of coercion or absence of coercion in over 70% of cases, which, however, also means it was unable to do so in almost 30% of cases as an important limitation requiring further research in order to avoid severe consequences in clinical practice. Furthermore, due to the retrospective nature of the present study, future research should focus on those parameters indicating a high risk of coercion before its occurrence, as only these parameters may become of clinical value in preventing coercion. As detailed in **Figure 2** in the *Results*, the most indicative parameters for coercion are also those becoming observable prior to the occurrence of coercion in the timeline of events. Despite all limitations, model performance measures indicated similar precision as was attained in the only two other studies (to our knowledge) also exploring direct coercive measures with machine learning (see **Table 5**) (15, 30), which seems satisfactory for the purpose of identifying patients vulnerable for direct coercion in order to provide for more timely, targeted, and effective preventive measures. However, some research proposes the inclusion of so-called predictor variables for coercion not related to patients, but to procedural (30, 68), architectural (69), and care team (70, 71) related factors to achieve even higher predictive power and goodness of fit.

**Table 5:** Comparison of this and prior studies on coercive measures employing machine learning.

	(30)	(15)	Current study
Topic of study	Predictors for direct coercive measures in patients with all diagnoses in general psychiatry	Predictors for mechanical restraint in patients with all diagnoses in general psychiatry	Predictors for direct coercive measures in patients with schizophrenia in forensic psychiatry
Sample studied	Patients with coercion: 170	Patients with mechanical restraint: 5050	Patients with coercion: 131
Data collection	Retrospective file content analysis	Retrospective health record and registry content analysis	Retrospective file content analysis
Number of potential predictors explored	Not specified	86	569
Similar predictor variables at statistical significance	Threat of violence as reason for involuntary admission^1^, prior involuntary admission to treatment, antipsychotic medication	Threat of violence measured with the Broset violence checklist, involuntary admission to treatment, threatening/abnormal behavior, sparse/non-coherent/non-informative verbal response	Threat of violence, coercive measures in prior treatment(s), haloperidol prescribed, daily olanzapine equivalent prescribed upon discharge, poor impulse control, hostility and uncooperativeness at admission, total PANSS-score at admission
Model accuracy (balanced)	66.5–78.5%	Not specified	73.3%
ROC AUC	0.73–0.75	0.87	0.8468
Sensitivity	60–69%	56%	72.87%
Specificity	78–83%	94%	73.68%

ROC AUC, receiver operating characteristic curve area under the curve method, a measure for the goodness of fit of a model (67); PANSS, Positive and Negative Symptom Scale.

^1^Authors see a limitation in their measuring threat of violence only in terms of reason for involuntary admission.

Adding credibility to findings of the present study, the most frequently identified parameters correlating with coercion in prior research were confirmed, including (threat of) violence (9, 18, 20, 24, 25, 36–40) and prior coercive measures (15, 17, 19, 22, 27, 30, 34, 35). Similarly, it may seem trivial that threat of violence, actual violence, the use of direct coercive measures, and an unfavorable legal prognosis upon discharge are correlated. Yet this also increases credibility of the findings presented here. New factors identified in the current study, seem to be hidden in broader categories in the only prior studies also exploring direct coercive measures with machine learning (**Figure 2**) (15, 30). This means, the present study adds important detail to current knowledge about factors correlating with direct coercion, for example, by identifying a specific antipsychotic and cumulated dosing in terms of daily olanzapine equivalent prescribed (instead of just antipsychotic medication) or specific patient behavior measures (poor impulse control, uncooperativeness, hostility, and total PANSS scores at admission instead of “abnormal behavior” in general). Most likely, the identification of this level of detail in variables correlating with coercion at similar accuracy and AUC as in prior research on patients with all diagnoses in general psychiatry (30) was enabled by the exploration of a larger set of variables in a therapeutic setting harboring patients at particular risk for coercive measures (3). However, it is important to note, the current study was conducted without external validation (64). Again, before any clinical decisions are to be based on the results presented here, future research needs to validate the identified model with a focus on variables indicating increased risk for coercion prior to its occurrence in other clinical populations. These populations should have no overlap with the one explored here and perhaps be less prone to the use of coercion and ideally be in different cultural and legal environments. Should factors occurring prior to coercion identified in the present study be confirmed, this would allow psychiatrists to identify patients at increased risk for experiencing coercion early on (at admission) and allocate resources (including measures targeting different key components to reduce coercion in high risk populations as identified by (72): leadership, training, post-seclusion and/or restraint review, patient involvement, prevention tools, and changes in the therapeutic environment) accordingly throughout inpatient treatment in aiming to reduce direct coercion with increased efficacy and at lower costs. This would not only reduce economic burdens to treatment facilities, but also emotional strain on patients and care teams. While sensitivity for the identification of patients at risk for experiencing coercion is substantial, clinicians should keep in mind that specificity is not 100%, so that the model (should it be confirmed in other populations of patients with schizophrenia) cannot identify all patients at risk. Additional clinical evaluations and improvement of model sensitivity and specificity in future research are needed in addition to a general discussion in the field on what margins of error would be considered to be ethically acceptable.

Somewhat resolving inconsistent results of prior studies, gender was not identified in the current study, which may however also be due to the small number of female patients in the sample studied here or in prior research. In this context it is interesting to note, that research exploring predictors for mechanical restraint in 5050 patients (31% female) in Denmark also could not identify gender as a significant predictor (15). On the topic of antipsychotics as a predictor variable, it should be noted that haloperidol is the standard antipsychotic used for involuntary medication and sedation in psychiatric emergency situations with acute threats of violence in Switzerland. The use of high cumulative antipsychotic dosages above the recommended maximum in the treatment of violent offender patients with schizophrenia has been noted elsewhere (73) and clinicians should critically review its usefulness. At the same time, more severe psychopathology in such patients, a predictor for coercion of itself in this and prior studies (15), may require higher antipsychotic dosing (73).

In addition, future research should address those limitations inherent to retrospective file analysis, including the use of a PANSS-adopted scale for content analysis of psychopathological data, which in some cases was recorded before the publication of that instrument. It should also take caution of selection effects due to data for this study stemming from only one forensic psychiatric institution in Switzerland (monocentric study) and a relevant subgroup of Swiss offender patients being unable to receive treatment in any forensic psychiatric facility due to their relative scarcity (74). Hence, future research should critically review results in different institutions and settings internationally. Similarly, it would be interesting to explore indirect coercive measures, which may be a substitute for direct coercive measures, just as seclusion, restraint, and involuntary medication seem to be substitutes for each other depending on legislation and cultural aspects (23, 63).

## Data Availability Statement

The raw data supporting the conclusions of this article will be made available by the authors, without undue reservation, to any qualified researcher.

## Ethics Statement

The study was reviewed and approved by the Cantonal Ethics committee of Zurich, Switzerland (Ref.-No. KEK-ZH-NR 2014-0480).

## Author Contributions

MG, JK, and SL designed the study and protocol. The survey of the data *via* questionnaire was preformed independently by both JK and SL. All statistical analyses were carried out by JK. The first draft of the manuscript was done by MG and JK. SL and MG edited multiple drafts and supervised the statistical analyses.

## Conflict of Interest

The authors declare that the research was conducted in the absence of any commercial or financial relationships that could be construed as a potential conflict of interest.
