# Supplementary material for: Identifying Direct Coercion in a High Risk Subgroup of Offender Patients With Schizophrenia via Machine Learning Algorithms
Source: Front Psychiatry. 2020 May 13;11:415. doi: 10.3389/fpsyt.2020.00415 (PMC7237713; doi:10.3389/fpsyt.2020.00415)
Supplement: Supplementary file 1 [file Table_1.docx]

Supplementary Materials

Default hyperparameters for model building during nested cross validation

| **Algorithm** | **Hyperparameter** |
| --- | --- |
| Logistic Regression | - |
| Tree | minisplit=20; cp=0.01; maxcomplete=4; maxsurrogate=5; usesurrogate=2; surrogatestyle=0; maxdepth=30;xval=10 |
| Random Forest | Ntree=500; replace=true; nodesize=1; importance=false; localImp=false; |
| Gradient Boosting | distribution=Bernoulli; n.tress=100; cvfolds=0; interaction.depth=1; n.minobsinnode=10; shrinkage=0.1; bag.fraction=0.5; train.fraction=1 |
| KNN | integer=7; numeric=2; logical=true |
| SVM | cost=1; nu=0.5; kernel=radial; degree=3; cachesize=40; tolerance=0.001; shrinking=true |
| Naive Bayes | laplace=0 |

Detailed performance results with confidence intervals (CI) for model building during nested cross validation

| **ML algorithm** | **Balanced**  **Accuracy (95% CI)** | **AUC (95% CI)** | **Sensitivity (95% CI)** | **Specificity (95% CI)** | **PPV (95% CI)** | **NPV (95% CI)** |
| --- | --- | --- | --- | --- | --- | --- |
| Logistic Regression | 75.13  (83.93-60.52) | 0.8462  (0.9678-0.7246) | 70.70  (84.84-51.63) | 79.57  (93.48-54.24) | 85.38  (95.38-65.15) | 61.68  (79.81-39.99) |
| Tree | 71.55  (81.63-57.02) | 0.7929  (0.9302-0.6556) | 76.43  (88.98-57.55) | 66.67  (85.40-41.58) | 79.47  (91.22-60.37) | 62.63  (82.05-38.65) |
| Random Forest | 74.12  (83.62-59.53) | 0.8577  (0.9753-0.7401) | 78.34  (90.29-59.59) | 69.89  (87.57-44.61) | 81.46  (92.50-62.54) | 65.66  (84.23-41.43) |
| Gradient Boosting | 72.63  (82.43-58.03) | 0.8374  (0.9619-0.7129) | 76.43  (88.98-57.55) | 68.82  (86.86-43.59) | 80.54  (91.97-61.38) | 63.37  (82.44-39.57) |
| KNN | 69.56  (79.82-54.92) | 0.7976  (0.9337-0.6615) | 68.15  (82.91-49.08) | 70.97  (88.28-45.64) | 79.85  (91.99-59.39) | 56.90  (76.34-35.30) |
| SVM | 76.85  (85.77-62.42) | 0.8393  (0.9632-0.7154) | 80.57  (91.78-62.00) | 73.12  (89.65-47.73) | 83.40  (93.76-64.85) | 69.04  (86.61-44.55) |
| Naive Bayes | 77.01  (85.78-62.47) | 0.8438  (0.9662-0.7214) | 85.35  (94.75-67.36) | 73.12  (89.65-47.73) | 84.28  (94.06-66.27) | 69.39  (86.88-44.82 |

Variance inflation factor (VIF) of multicollinearity test

| R13a | 1.492796 |
| --- | --- |
| PH12a | 1.528415 |
| R8a | 1.804363 |
| R20a | 1.562970 |
| PANSSH22 | 1.883720 |
| R9e | 1.353832 |
| PANSSSCOREADMH | 2.039396 |
| PANSSH7 | 2.348723 |
| R28 | 2.983519 |
| PANSSH28 | 1.869285 |
